# Inhibitory Effects of Plant Trypsin Inhibitors *Msti-94* and *Msti-16* on *Therioaphis trifolii* (Monell) (Homoptera: Aphididae) in Alfalfa

**DOI:** 10.3390/insects10060154

**Published:** 2019-05-30

**Authors:** Hailong Zhao, Hidayat Ullah, Mark Richard McNeill, Guilin Du, Kun Hao, Xiongbing Tu, Zehua Zhang

**Affiliations:** 1 State Key Laboratory for Biology of Plant Diseases and Insect Pests, Institute of Plant Protection, Chinese Academy of Agricultural Sciences, Beijing 100193, China; zhao.hailong@live.com (H.Z.); shabkadar@yahoo.com (H.U.); haokun8611@foxmail.com (K.H.); 2 College of Plant Protection, Shenyang Agricultural University, Shenyang 110161, China; 3 Department of Agriculture, The University of Swabi, Anbar-23561, Swabi, Khyber Pakhtunkhwa, Pakistan; 4 AgResearch, Lincoln Research Centre, Christchurch 8140, New Zealand; mark.mcneill@agresearch.co.nz; 5 National Animal Husbandry Service, Beijing 100125, China; caasdgl@163.com

**Keywords:** enzyme activity, reproduction, spotted alfalfa aphid, survival, trypsin inhibitor

## Abstract

The spotted alfalfa aphid (*Therioaphis trifolii* (Monell)) is a known destructive pest that can significantly reduce alfalfa yields. Two differentially up-regulated alfalfa trypsin inhibitors ‘*Msti-94*’ and ‘*Msti-16*’ in transcriptome were verified in terms of their mRNA levels using RT-qPCR. The prokaryotic expression vector was constructed and its biological functions, including phenotypic and physiological responses, were verified through feeding spotted alfalfa aphids with active recombinant protein mixed with an artificial diet. Gene clone and gene prokaryotic expression confirmed that *Msti-94* had a size of 651 bp, encoded 216 amino acids with a predicted protein weight of 23.5 kDa, and a pI value of 6.91. Similarly, the size of *Msti-16* was 612 bp, encoded 203 amino acids, and had a predicted protein weight of 22.2 kDa with a pI value of 9.06. We concluded that both *Msti-94* and *Msti-16* acted as a stomach poison with survival rates reduced to 21.7% and 18.3%, respectively, as compared to the control, where the survival rate was significantly (*p* < 0.05) higher (60.0%). Aphid reproduction rates were significantly reduced, after 72 h of feeding, in both the *Msti-94* and *Msti-16* treatments compared to the controls. A concentration of 800 μg/mL (0.8 mg/mL) of recombinant protein and 5000 μg/mL (5 mg/mL) of recombinant expressing bacteria that inhibits the total protease, which ultimately disrupted the activity of trypsin, chymotrypsin, and aminopeptidase.

## 1. Introduction

*Therioaphis trifolii* (Monel) (Homoptera: Aphididae), the spotted alfalfa/clover aphid, often gathers on the lower parts of leaves and tender buds, where its prickly mouthpiece pierces the plant tissue and feeds on plant juice, causing the discoloration and yellowing of leaves. This ultimately restricts plant growth, causing senescence, referred to as the “green vein syndrome” [1,2]. Feeding can cause yield losses ranging from 20 to 30% [3]. The spotted alfalfa aphid was first reported in New Mexico, United States of America, where it had caused the wide-scale destruction of alfalfa crops [4]. The aphid is also found in the regions of Hebei, Shandong, Henan, Shanxi, Gansu, Yunnan, Jilin, Liaoning, the Beijing municipality, and Mongolia, where infestations have been shown to cause an overall economic value reduction in more than 60% of alfalfa crops [5].

Alfalfa (*Medicago sativa* L.) also known as “the King of forage” [6] is prone to serious damage from a range of pests, including aphids, thrips, leaf mining diptera, and other host-specific pests [7,8]. Plant protease inhibitors (PIs) naturally occur in plants, and they can act as a defense against insect herbivory [9]. PIs bind to trypsin in the insect gut to form enzyme inhibitor complexes that affect the synthesis and regulation of alimentary proteases, thereby disrupting the digestion and absorption of nutrients [9]. They affect the synthesis and regulation of intestinal protease, which ultimately inhibits the digestion and absorption of nutrients in insects by binding with trypsin to form an enzyme inhibitor (EI) complex [10]. Certain insects and many of the phytopathogenic microorganisms secrete extracellular enzymes and, in particular, enzymes that are responsible for the proteolytic digestion of proteins, which play important roles in pathogenesis [11]. At present, a variety of plant PI genes have been developed and applied independently in agricultural technology production to improve plant defense against insect herbivores. The cowpea trypsin inhibitor (CpTI) was successfully transformed into tobacco for the first time, and it has been confirmed along with the *Bacillus thuringiensis* (Bt) gene [12,13]. Similarly, a substance from soybean was isolated and it proved to be a plant PI [14]. The transgenic plants successfully developed a resistance to *Heliothis*, *Spodoptera*, *Diabrotica*, and *Locusta*, citing further research involving 15 different PIs that were transferred into plants to produce insect-resistant lines [15,16,17,18,19].

Trypsin inhibitors (TI) and chymotrypsin inhibitors (CTP) are shown to significantly impair the growth of Lepidopteran larvae in field crops [20], whilst the cysteine proteinase inhibitor (CPI) inhibits the growth and development of *Coleoptera* larvae by disturbing the digestive system of insects as a target region for pest control [21]. The hydrolytic activity of most proteases in the intestine of the beet armyworm (*Spodoptera exigua*) and the American cotton bollworm (*Helicoverpa*) was controlled using soybean trypsin inhibitors (STI) [22]. The application of the STI at 50 μg/mL (0.05 mg/mL) inactivated around 88% of the trypsin in the midgut of *Prodenia litura* (*Spodoptera litura*) [23]. Similarly, by increasing the concentration of mustard chymostatin 8 (Chy8) to 500 μg/mL (0.5 mg/mL), the death rate of Chy8 was 100% in 72 h, and the death rate of mustard trypsin inhibitor-2 (MTI-2) also increased to 100% in 144 h for soybean aphids [24]. Previously, transcriptome analyses revealed the Zhongmu-1 (resistant) and Soca (susceptible) levels of resistance to aphids using the feeding method over 72 h [25].

To investigate the potential of the PI in controlling the spotted alfalfa aphid, the current study was designed with two differentially up-expressed *Medicago sativa* trypsin inhibitor (Msti) genes. They were validated using the quantitative real-time polymerase chain reaction (RT-qPCR) and cloned by means of the molecular biological technique. The prokaryotic expression vector was constructed and their biological, phenotypic, and physiological functions were verified through feeding experiments followed by enzyme-linked immunosorbent assay (ELISA) testing [26]. This provided a good theoretical basis for the development and utilization of new insect-resistant gene resources.

## 2. Materials and Methods

### 2.1. Aphid Sample

Adult spotted alfalfa aphids were primarily collected from an alfalfa research site at the Hebei Academy of Agricultural Sciences in Cangzhou, Hebei Province, where they were augmented from a culture held by the China Agricultural University, Beijing. The aphids were transferred to glasshouse grown alfalfa (cv. Zhongmu-1 (resistant line) and WL323 (susceptible line), and then maintained over approximately three generations in a controlled environment chamber at 25 ± 1 °C, 75% RH, and a 16:8 L:D photoperiod.

Prior to commencing the experiment, healthy and energetic active 3rd–4th instars were selected from the colony, and >200 aphids were placed in a sterilized 9 cm plastic petri dish. Subsequently, each culture dish received more than 60 aphids per repetition in a controlled environment chamber, and then starved for 12 h prior to commencement of the experiment.

### 2.2. Artificial Feeding Device and Artificial Diet

Based on the method of the double-layer Parafilm M^®^ clip liquid artificial recommended feed [27], a transparent glass tube with a diameter of 2~3 cm and a height of 4~5 cm was used with a slight modification in the diameter of the container, the external pressure applied, and sucrose quantity [27,28] (Figure S1). On its top, the feed storage and activity room had a double-layer Parafilm M^®^ “sachet shape”. The bottom was ventilated with a sponge or gauze seal. The Parafilm M^®^ was treated with germicide for several minutes to provide an aseptic environment prior to starting the operation. To avoid any spoilage of diet during the manipulations, the operators wore disposable gloves.

Artificial feed (diet) was modified from the suggested formula [28] for the aphids by adding honey and increasing the concentration of folic acid and saccharose (Table 1). The diet mixture was stirred consistently using a magnetic agitator along with a bacterial filter of 0.45 microns, and then stored at −20 °C to avoid thawing and freezing. The aphids were successfully fed for several generations.

### 2.3. Alfalfa Sample

Seeds from two of the selected varieties (cv. Zhongmu-1 and cv. WL323) of alfalfa were obtained from Professor Yang’s laboratory, China Agricultural University. The seeds were sown in pots and grown in a controlled environment chamber at a temperature of 27 ± 2 °C, at a relative humidity of 75%, and a 16:8 L:D photoperiod.

### 2.4. Verification of the mRNA Level Using RT-qPCR

Thereafter, in the treated experiments, two groups of 30 aphids were allowed to feed on one of the two cultivars of alfalfa for 72 h. The control plants were not exposed to the aphids and there was no introduction of aphids for the same period. There were five replicates per treatment, creating a total of 20 plants. Then the total ribonucleic acids (RNAs) of the two alfalfa cultivars with and without aphids were extracted, and the residual deoxyribonucleic acid (DNA) was digested with DNase (Deoxyribonuclease I) (TaKaRa, Dalian, China). The earliest strand of complementary DNA (cDNA) was synthesized using the Prime Script^TM^ ‘1st strand cDNA Kit (TaKaRa, Dalian, China). The concentration of cDNA was measured using a Nano Photometer (IMPLEN) and then cryopreserved at −80 °C. The differentially expressed genes were analyzed at the messenger RNA (mRNA) level using the SYBR Green fluorescent quantitative kit (TaKaRa, Dalian, China), with 18 s of RNA as an internal reference gene through RT-qPCR. The program includes three steps: the first step was pre-denaturation at 95 °C, for 3 min; the second step included denaturation at 95 °C, for 15 s; and the third step was annealing at 60 °C, for 1 min. This process was repeated for 40 cycles. Specific primers for the RT-qPCR of *Msti* are shown in Table 2.

### 2.5. Electrophoresis of the Prokaryotic Expression and Protein Purification

Gene sequences of *Msti-94* and *Msti-16* from the transcriptome of alfalfa were selected for the aphids, and their putative protein sequences are described in Table 3 and Table 4. Specific genes were cloned and the recombinant express vectors ‘pS-*Msti-94*’ and ‘pS-*Msti-16*’ were constructed through the prokaryotic expression vector pSYNO-1 (Suzhou Hongxun Biotechnologies Co. Ltd., Suzhou, China), and then transformed into BL21-DE3 (TaKaRa, Dalian, China) competent cells by adding isopropyl-β-D-thiogalactopyranoside (IPTG) to the final concentration of 1 mM at 37 °C for 3 h. For protein expression, the recombinant bacteria were broken down with a buffer comprising 20 mM phosphate-buffered saline (PBS) and 150 mM NaCl at 7.4 pH, and then detected by sodium dodecyl sulfate–polyacrylamide gel electrophoresis (SDS-PAGE) in 10% SDS polyacrylamide gel (Table 5). The protein purification process was performed via affinity chromatography using a nickel column with an equilibrium solution (20 mM Tris, 500 mM NaCl, pH 8.0), that was washed by an imidazole eluent buffer with concentrations of 100 mM, 200 mM, and 500 mM at a pH level of 8.0 to standardize the final feeding product. Similarly, to purify the protein, centrifugal ultrafiltration (Amicon^®^ Ultra line centrifugal filter tube; EMD Millipore portfolio, Massachusetts, MA, USA; 30 kDa) was used to minimize the toxic effect of salt and imidazole. Protein concentration was determined using a NanoDrop ultramicro spectrophotometer at A260/A280 and then confirmed through a Bradford assay (Synbio Technologies, Jiangsu-China). Similarly, the purity and size of the recombinant protein was roughly determined by the number and gray level of bands in the SDS-PAGE. The determined purity of the recombinant protein was estimated as ≥90%.

### 2.6. Biological Assay of Msti the on Spotted Alfalfa Aphid

#### 2.6.1. Recombinant Expressing Bacteria

Large numbers of recombinant expressing bacteria were centrifuged at 10,000× *g*, and suspended with normal saline broken by an ultrasonic centrifuge. The unbroken cells and the precipitates were eliminated after proper centrifugation. Using 0.45 microns of bacterial filter, the collected supernatant was filtered. All the operations were carried out in sterilized conditions to minimize the establishment and growth of bacteria. The supernatant was dried into powder using a vacuum freeze dryer to avoid the denaturation of proteins. Equilibrium solution (20 mM Tris, 500 mM NaCl, pH 8.0) and centrifugal ultrafiltration (Amicon^®^ Ultra line centrifugal filter tube; EMD Millipore portfolio, MA, USA; 30 kDa) were used to minimize the toxic effect of salt and imidazole. Firstly, the EMD Millipore portfolio was used to perform the recombination of the proteins without salts and imidazole with PBS buffer, and then it was used to suspend the protein. The protein concentration was determined using a NanoDrop ultramicro spectrophotometer at A260/A280, and the confirmed through a Bradford assay (Synbio Technologies, Jiangsu-China), followed by purity and size determination through SDS-PAGE via the number and gray levels of bands. The determined purity of the recombinant protein was estimated as ≥90%. In addition, there may have been other proteins. However, as the total recombinant protein quantity was comparatively high (90%), we assumed that the effect of these other proteins would not be significant. The methodology in Reference [29] was used to prepare the supernatant concentration of 2000, 3000, 4000, 5000, and 6000 μg/mL using PBS suspension buffer. Unexpressed bacteria were not induced to deal with the same treatment. Then, the supernatant of different concentrations of induced recombinant expression bacteria were used as the treatment group for the bioassay, and the unexpressed bacteria’s solution was kept the same as that of the control group. Thereafter, four-day old adult aphids were randomly selected using an artist’s fine paintbrush moistened with tap water, wherein 10 aphids were added to each feeding device. Each gene expression bacteria was divided into five treatment groups along with five control groups, with six repetitions per group to give a total of 60 devices.

#### 2.6.2. Recombinant Purified Protein Treatment

The expressed *Msti* recombinant purified protein was diluted to 800 μg/mL with ultrapure water and then preserved at −20 °C as described in Reference [29]. Then, four-day old adult aphids were randomly selected using an artist’s fine paintbrush moistened with tap water. We setup two treatment groups and one control group containing 10 aphids per feeding device, with six replicates per treatment.

#### 2.6.3. Feeding Method

The primary layer of Parafilm M^®^ was used to seal the feeder followed by the inclusion of 100 μL of recombinant *Msti*, followed by a secondary layer of Parafilm M^®^ that was used to preserve the moisture. The aphid device was then transferred to a controlled environment chamber set at a temperature of 25 ± 1 °C, 75% relative humidity, a 16:8 L:D photoperiod, and a light intensity of 9230 lux. The bioassay ran for 72 h, with aphid mortality recorded every 24 h where dead individuals were removed, and the reproduction rate was recorded once after 72 h. Graphpad-prism5 software was used to visualize the mortality and reproduction curves.

### 2.7. Pre-Treatment of Enzymatic Activities of Samples

At the end of the 72 h bioassay, the surviving aphids were removed and ground in PBS buffer at 2 μL per aphid, and then exposed to a lapping powder shape with a liquid nitrogen cryogenic ice bath at 12,000 × *g* for 10 min at 4 °C by eliminating the impurity and retaining the supernatant. The entire process was repeated twice. The crude extract of the total protein was prepared, and we used the same amount of PBS buffer for the control group as well. The equation for the different enzyme curves is given in Table S1, along with the regression values.

### 2.8. Determining Total Enzymatic Activity

The determination of total protease (Azocasin 20 mg/mL as substrate), trypsin-*N*-α-benzoyl-L-arginine ρ-nitroanilide (BApNA 1 mg/mL as substrate), chymotrypsin (BETT 1 mg/mL as substrate), and aminopeptidase (L-Leu-pNA 1 mg/mL as substrate) activity was carried out as per specified protocols in References [17,30,31,32], using an ELISA-sandwich technique kit (Affandi Co. Ltd., Shanghai, China). We took fifty microliters of different concentrations of samples from each standard, then treated it with 100 μL of horseradish peroxidase (HRP) to label the antibodies [26], and then sealed it at 37 °C in a water bath for 60 min, followed by the addition of 50 μL substrate in the dark at 37 °C for an additional 15 min. The sample’s optical density (OD) value of 450 nm was measured after addition of the termination solution.

## 3. Results

### 3.1. Verification of the mRNA Level Using RT-qPCR

**Figure 1:**
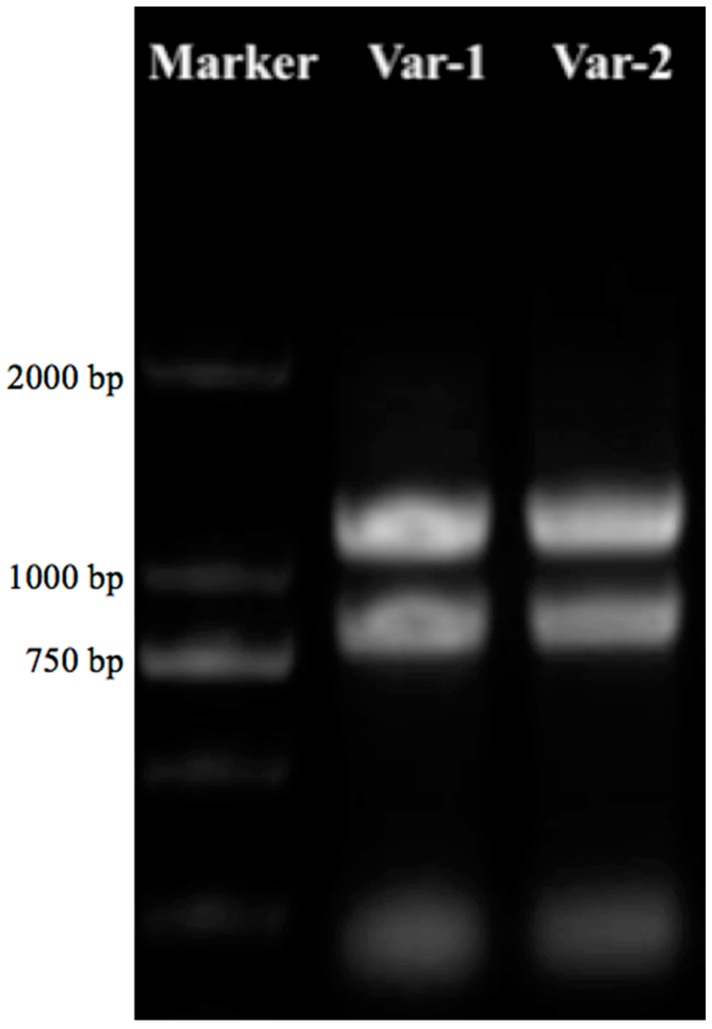
Clear bands of the RNA for alfalfa confirmed by RT-qPCR, where lane 1 represents the marker (DL2000), lane 2 the Var-1 resistance variety (Zhongmu-1), and lane 3 the Var-2 sensitive variety (WL323).

Using RT-qPCR, the RNA was extracted from the freshly picked leaves of two selected cultivars of alfalfa, which clearly showed the 5S, 18S, and 28S bands of RNA (Figure 1).

**Figure 2:**
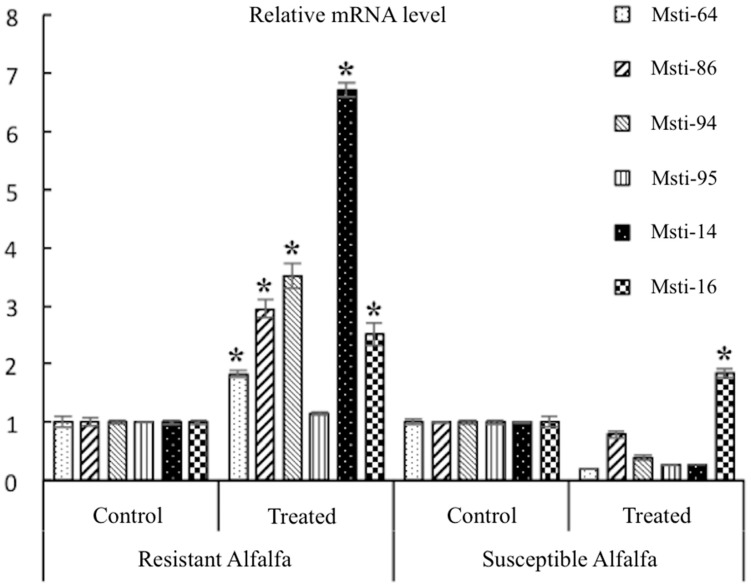
Relative expression of the six selected differentially expressed genes (DEGs), where control refers to treatments without aphids after 72 h, and treated refers to treatments with 30 aphids feeding after 72 h on resistant (cv. Zhongmu-1) or susceptible (WL323) alfalfa cultivars; * significant at a 5% level of probability using SPSS 20.0 with the Duncan multiple range test.

RT-qPCR analysis confirmed that the six selected differentially expressed genes (DEGs)—i.e., Kunitz-type trypsin inhibitor (*Msti-64*); Serine proteinase inhibitor (*Msti-86*); Kunitz-type trypsin inhibitor-like 2 protein (*Msti-94*); Kunitz-type trypsin inhibitor/Alpha-fucosidase (*Msti-95*); and alpha-amylase/subtilisin inhibitor (*Msti-14*)—had consistent expressions in a manner similar to that obtained by transcriptomes at the transcriptional level. Gene expressions were up-regulated in the resistant alfalfa variety (cv. Zhongmu-1) after feeding by the aphids, whilst the expressions were down-regulated in the sensitive variety of alfalfa (cv. WL323). The level of gene expression was significantly (*p* ≤ 0.05) higher in the treatments with aphids as compared to the treatment without aphids (Figure 2). Similarly, the expression was significantly higher in the resistant varieties of alfalfa as compared to the susceptible varieties.

### 3.2. Gene Cloning, Prokaryotic Expression, and Protein Purification of Msti

**Figure 3:**
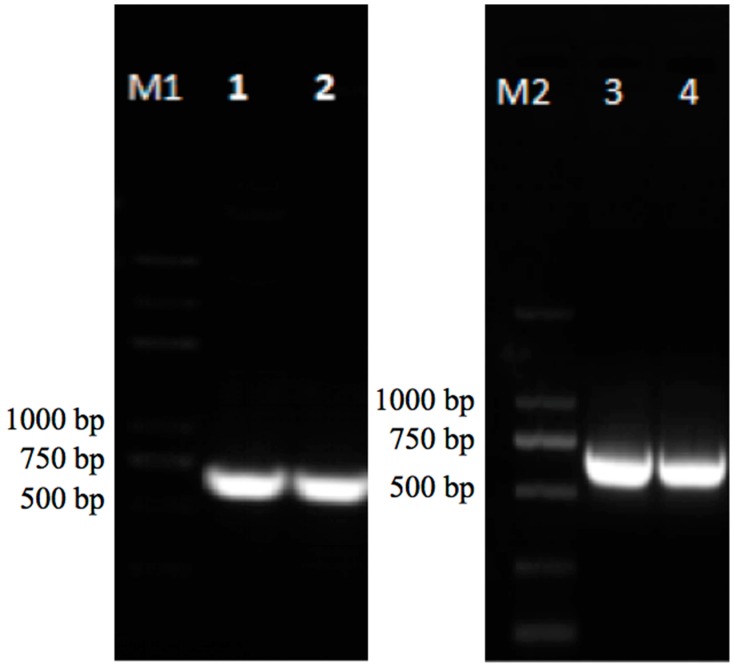
Agarose gel electrophoresis of primer specificity in the amplification of genes. M1 and M2: DL2000 plus marker (TaKaRa); Lane 1–2: *Msti-94* (Zhongmu-1); and *Msti-94* (WL323), respectively; Lane 3–4: *Msti-16* (Zhongmu-1) and *Msti-16* (WL323), respectively.

Two of the selected candidate genes (*Msti-94* and *Msti-16*) were successfully cloned using specific primers of *Msti-94* (5′GGATCC**ATG**AAGCATCTTTTATCACTA3′) and *Msti-16* (5′GGATCC**ATG**AAAACCTCACTCTTAGC3′) on two selected verities of alfalfa (Figure 3). *Msti-94* was 651 bp and encoded 216 amino acids, with a predicted protein weight of 23.5 kDa and a pI value of 6.91. Similarly, *Msti-16* was 612 bp and encoded 203 amino acids, with a predicted protein weight of 22.2 kDa and a pI value of 9.06. The sequence homology with *Medicago truncatula* L. (barrel medick) trypsin inhibitors reported in GenBank was over 95% (Table 6). Although *Msti-64*, *Msti-86*, and *Msti-14* were also significant and had potentially vital roles, they were still omitted from further analysis.

**Figure 4:**
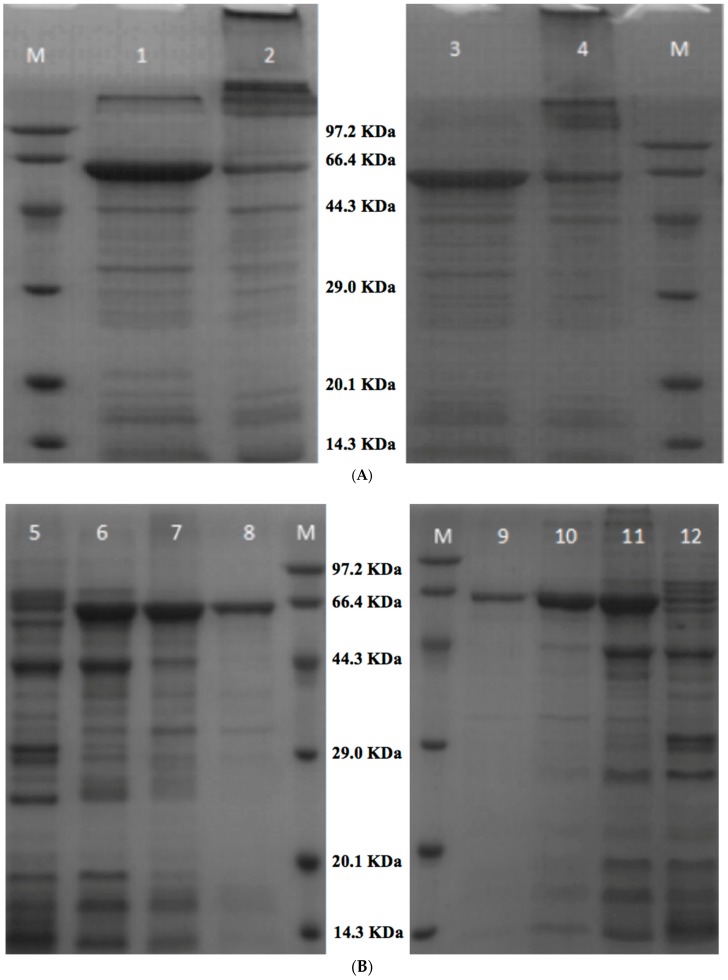
SDS-PAGE analysis of the prokaryotic-expressed target protein in 10% SDS polyacrylamide gel. (**A**) M: 100 kDa protein marker (Transgene); Lane 1: *Msti-94* reduced protein, Lane 2: *Msti-94* non-reduced protein, Lane 3: *Msti-16* reduced protein, Lane 4: *Msti-16* non-reduced protein; (**B**) Lane 5–8: *Msti-94* expression protein elution via 50 mM, 100 mM, 200 mM, 500 mM imidazole, respectively; M: 100 kDa protein marker (Transgene); Lane 9–12: *Msti-16* protein expression elution using 500 mM, 100 mM, 200 mM, and 50 mM imidazole, respectively; Isopropyl β-D-1-thiogalactopyranoside (IPTG) at the final concentration of 1 mM, induction at 37 °C for 3 h; non-reduced without IPTG; breaking the bacteria with a buffer of 20 mM PBS, 150 mM NaCl, pH 7.4.

The recombinant expression vectors pS-*Msti-94* and pS-*Msti-16*, containing BamH I and Xhol I (TaKaRa) restriction sites, were respectively constructed through double enzyme digestion with the MBP, GST, and His tag (where the His-tag was used for protein purification and Ni-column binding, whilst the GST and MBP tags were designed to enhance the protein solubility and screening for the effectiveness of toxic proteins). Respectively, the predicted mol masses of the *Msti-94* and *Msti-16* recombinant proteins were 65.5 kDa and 64.2 kDa. PAGE-SDS electrophoresis on 0.5 mM IPTG confirmed the weight of *Msti-94* (23.5 kDa) and *Msti-16* (22.2 kDa), where the label size of the vector used was 42.0 kDa (Figure 4A,B). The best elution effect was obtained using 500 mM imidazole (Figure 4B, lane-8 and lane-9) to finalize the feeding experiment. Since the effect of purification was better, and the tags on the expression vector pSYNO-1 had no subsequent effect on the feeding experiment, we directly used the purified protein with a tag for the follow-up experiments.

### 3.3. Analysis of Survival and Reproduction

#### 3.3.1. Effect of the *Msti* Recombinant Expressing Bacteria of Spotted Alfalfa Aphids

**Figure 5:**
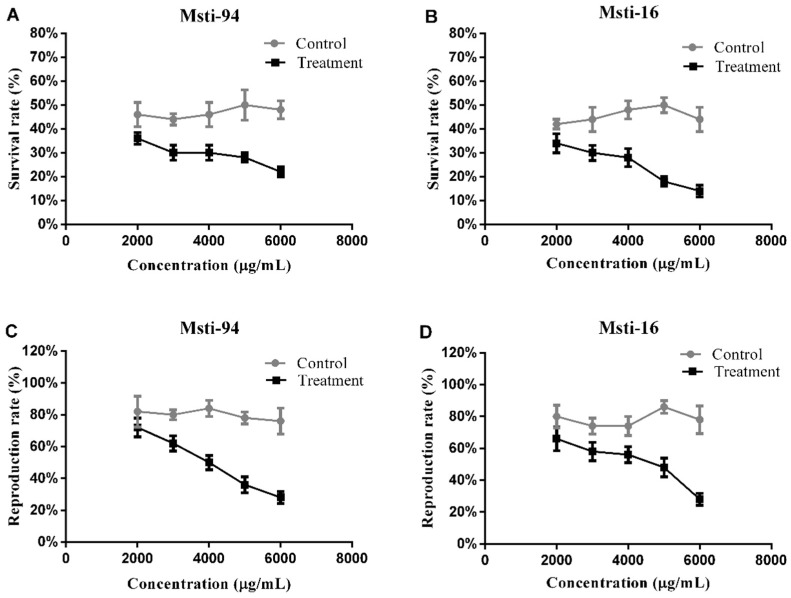
The survival rates of the aphids after 72 h at different concentrations of feeding with *Msti-94* (**A**); survival rates of the aphids after 72 h at different concentrations of feeding with *Msti-16* (**B**); reproduction rates of the aphids after 72 h at different concentrations of feeding with *Msti-94* (**C**); the reproduction rates of the aphids after 72 h at different concentrations of feeding with *Msti-16* (**D**). Control refers to the artificial feed without any inhibitors (non-induced bacterial sample), and treatment refers to the artificial feed with inhibitors.

The survival rate of aphids feeding on the *Msti* recombinant expressing bacteria declined with an increased concentration, when measured at the end of the bioassay (72 h). In the treatment experiment, survival from *Msti-94* was 36, 30, 30, 28, and 22% at concentrations of 2000, 3000, 4000, 5000, and 6000 μg/mL, respectively, which was significantly lower (*p* < 0.05) compared to the control group (Figure 5A) at high concentrations (≥3000 μg/mL). Similarly, in the treated experiment, the percentage of survival of aphids from *Msti-16* was 34, 30, 28, 18, and 14% at concentrations of 2000, 3000, 4000, 5000, and 6000 μg/mL, respectively, which was significantly lower (*p* < 0.05) compared to the control group (Figure 5B) at a concentration of ≥3000 μg/mL.

In a similar manner, the reproduction rate of aphids decreased with the increase in concentration after feeding for 72 h. The reproduction rates of the aphids treated with *Msti-94* was recorded as 72, 62, 50, 36, and 28% at concentrations of 2000, 3000, 4000, 5000, and 6000 μg/mL, respectively, which was significantly lower (*p* < 0.05) than the control group (Figure 5C) at high concentrations (≥3000 μg/mL). Similarly, the reproduction rate was 66, 58, 56, 48, and 28% at concentrations of 2000, 3000, 4000, 5000, and 6000 μg/mL for the treatment with *Msti-16*, respectively, which was also significantly lower (*p* < 0.05) than the control group (Figure 5D) at high concentrations (≥3000 μg/mL).

#### 3.3.2. Effect of the *Msti* Recombinant Purified Protein on the Spotted Alfalfa Aphids

**Figure 6:**
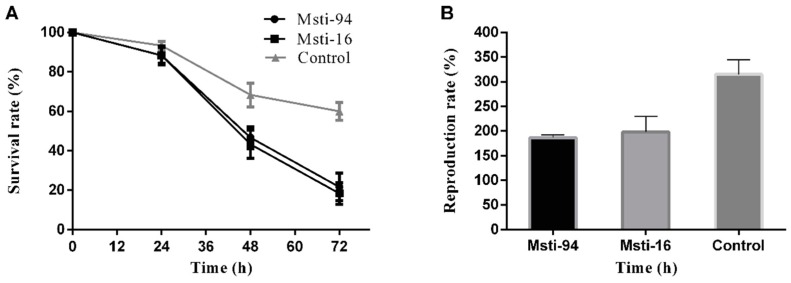
Control had artificial feed without any inhibitors (non-induced bacterial sample) (**A**) The survival rate of aphids fed for 72 h with *Msti*, with a treated dose of 800 μg/mL purified protein; (**B**) reproduction rate of aphids fed for 72 h with *Msti*, with a treated dose of 800 μg/mL purified protein. The reproduction rate of the aphids was higher than 100% because of parthenogenesis and overlapping generations.

*Msti* recombinant purified protein determinations in the aphids showed that the survival rate was reduced within 72 h after feeding on 800 μg/mL of both the *Msti-94* and *Msti-16* recombinant purified proteins. After 72 h of feeding, the recorded survival rate was 21.7% (*F* = 16.264, *p* = 0.04) and 18.3% (*F* = 26.334, *p* = 0.03) for *Msti-94* and *Msti-16*, respectively (Figure 6A). The survival rate of aphids feeding on *Msti-16* was comparatively lower than that of *Msti-94*, whilst the aphid survival rates for both of them were lower than that of the control group by 60.0% (*F* = 6.194, *p* = 0.025).

Feeding aphids with *Msti-94* and *Msti-16* recombinant purified protein also affected the reproduction rates. The rate of reproduction of aphids fed with *Msti-94* and *Msti-16* recombinant purified proteins decreased within 72 h by 186.7% (*F* = 7.929, *p* = 0.04) and 198.3% (*F* = 10.714, *p* = 0.05), respectively. The reproduction rate of aphids on *Msti-94* was comparatively lower (186.7%; *F* = 7.929; *p* = 0.04) than *Msti-16* (198.3%; *F* = 10.714; *p* = 0.05). However, both of the feeds were significantly lower (Figure 6B) than the control group (315.0%; *F* = 5.143; *p* = 0.039).

### 3.4. Enzymatic Activities

#### 3.4.1. Effect of the *Msti* Recombinant Expressing Bacteria on Enzyme Activity in Spotted Alfalfa Aphids

**Figure 7:**
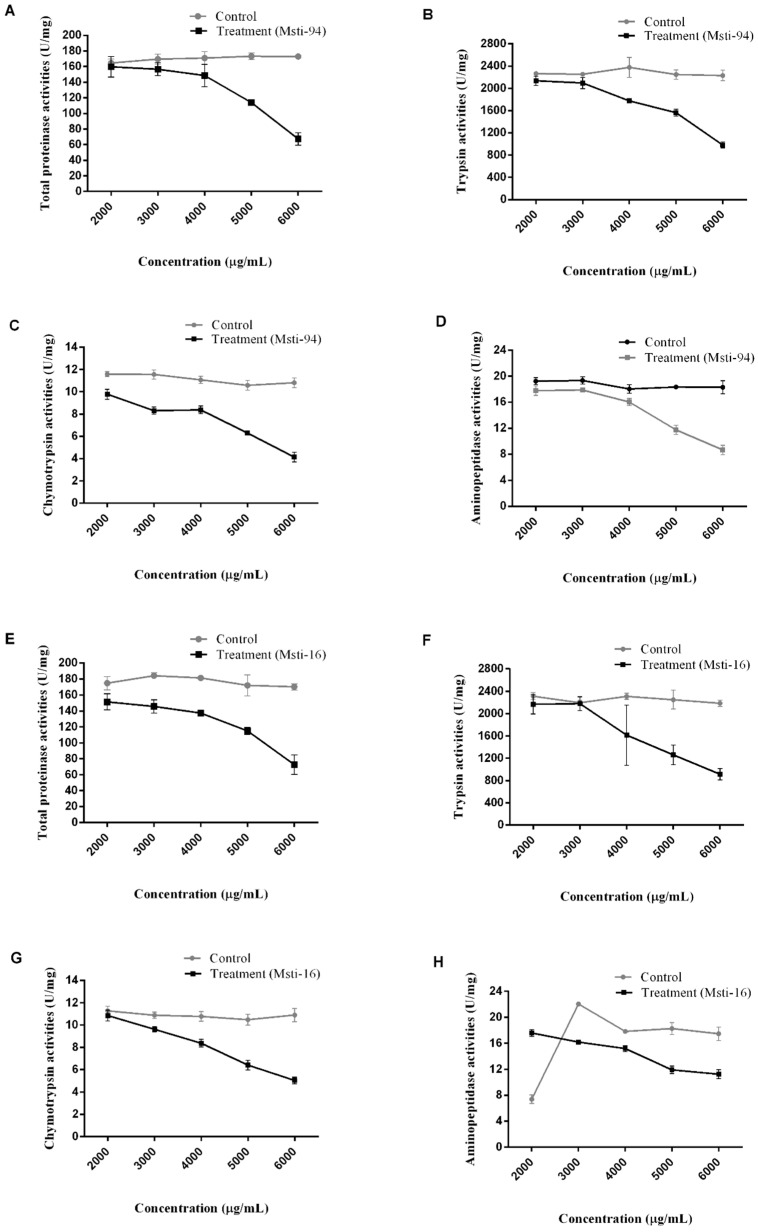
Activities of the four selected enzymes treated with different concentrations of *Msti-94* and *Msti-16* added to the artificial feed along with the control. Total proteinase vs. *Msti-94* (**A**); trypsin vs. *Msti-94* (**B**); chymotrypsin vs. *Msti-94* (**C**); aminopeptidase vs. *Msti-94* (**D**); proteinase vs. *Msti-16* (**E**); trypsin vs. *Msti-16* (**F**); chymotrypsin vs. *Msti-16* (**G**); aminopeptidase vs. *Msti-16* (**H**). The calculation method of the enzyme activity is based on the international calculation formula, and an enzyme activity unit (U) is defined as the reduction or production of 1 μmol substrate in 1 min. Control refers to the group with the artificial feed without any inhibitors (non-induced bacterial sample), and treatment refers to the artificial feed with inhibitors.

‘Huaao Biological’ enzymatic activities showed that the total proteinase, trypsin, chymotrypsin, and aminopeptidase in the aphids were hindered after the aphids were treated with both *Msti* recombinant-expressing bacteria’s at different concentrations (2000, 3000, 4000, 5000, and 6000 μg/mL). The activity of the studied four enzymes was significantly (*p* < 0.05) reduced for *Msti-94* concentrations above 4000 μg/mL (Figure 7A–D; Table S2). In a similar manner, the enzyme activities of the protease, trypsin, chymotrypsin, and aminopeptidase were significantly (*p* < 0.05) halted at a concentration above 4000 μg/mL in the aphids treated with *Msti-16* (Figure 7E–H; Table S3). Initially, the aminopeptidase activity was comparatively lower and unexpressed; however, it increased with the 3000 μg/mL treatment, and then it was reduced and inhibited with enhanced concentrations. Enhanced activity of aminopeptidase might be associated with the proteolytic activities of other enzymes.

#### 3.4.2. Effect of the *Msti* Recombinant Purified Protein on Enzyme Activity in Spotted Alfalfa Aphids

**Figure 8:**
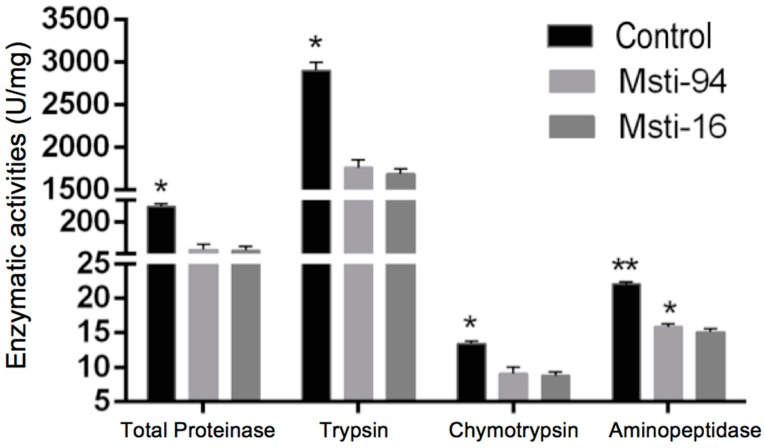
Enzyme activities of the four protease in aphids treated with *Msti* along with the control. *Msti-94*/*Msti-16*: the recombinant purified protein of *Msti* at a concentration of 800 μg/mL was used to feed the aphids. Control: the artificial feed was used to feed the aphids. *, ** represents the level of significance at 5% and 10%, respectively.

A decrease in the enzyme activities of total proteinase, trypsin, chymotrypsin, and aminopeptidase was observed at a concentration of 800 μg/mL of *Msti* recombinant purified protein in the spotted alfalfa aphids (Figure 8; Table S4). The total protease activity of aphids was significantly (*p* < 0.05) decreased by 41.0% and 41.8% when it was treated with the *Msti-94* and *Msti-16*, respectively. Similarly, the activities of trypsin, chymotrypsin, and aminopeptidase were also significantly (*p* < 0.05) decreased by 39.3%, 41.9%, and 32.0%, respectively, when they were treated with *Msti-94*, whilst the activity decreased by 34.4%, 28.1%, and 31.5%, respectively, when treated with *Msti-16*.

## 4. Discussion

Plant protease inhibitors are a class of peptides or proteins that are known for their proteolysis activities [33]. This group or family has many sub-families, wherein the serine protease inhibitors are the most widely studied. In this experiment, Kunitz-type trypsin inhibitors (KTI) were studied, where they have been previously reported for soybean [34]. These inhibitors had a molecular weight of about 20 kDa. Few studies on the insecticidal activity of these STI against aphids are documented. In this paper, the inhibitory effects of two *Msti* on the spotted alfalfa aphid were determined by feeding the aphids an artificial diet to determine more effective botanical pesticides and provide a scientific basis for the integrated management of alfalfa pests. We found that *Msti* recombinant purified protein may inhibit the density of aphids at a specific concentration. The KTI family has an inhibitory specificity, containing a single reaction site and a functional inhibitory domain that specifically affects trypsin [35]. *Msti* might have inhibited chymotrypsin by destroying the digestive system of the aphids, which created a contrasting environment for the expression of other elementary enzymes. A number of proteases in the insect digestive system have been reported. Insects usually depend on other proteases to maintain normal growth and the development of one of the proteases inhibits its developmental activity. Simultaneously inhibiting several types of proteases will significantly affect biological function [36].

Phytoprotease inhibitors have been studied to understand the basis of insect resistance in several crops [37], and in China, they have become the main mode of crop pest control with several bio-pesticides being formulated using this approach [38]. The main available and studied phytoproteases in certain plants for understanding insect pest resistance, are SKTI and the Bowman–Birk inhibitor (BBI) [39]. Using SKTI at a concentration of 500 μg/mL led to the death of the larvae of *Anthonomus grandis* (cotton boll weevil) as the growth and development of hatched larvae was stopped [40]. In our research, we found that *Msti* in alfalfa affected the survival and reproduction of spotted alfalfa aphids, and it ultimately caused 100% mortality at a concentration of 800 μg/mL. On the other hand, the application of SKTI on cabbage moth (*Mamestra brassicae*) larvae revealed that it had no significant effect on mortality; but it significantly inhibited the moth’s gain in body weight [41]. Previous studies have also revealed that trypsin and chymotrypsin inhibitors can inhibit Lepidoptera larvae, whilst cysteine protease inhibitors retarded the growth of *Coleoptera* larvae [20,21]. We also observed that the inhibition of trypsin and trypsin-like enzymes reduced general proteolysis in the aphid gut, which might affect the expression and activity of amino peptidase. Therefore, plant protease inhibitors can be used as potential plant derivative pesticides for the control of noxious pests in crop plants.

The survival rates of spotted alfalfa aphid were reduced by 21.7% and 18.3%, respectively, compared to the control when aphids were treated with *Msti-94* and *Msti-16* in the selected alfalfa cultivars. These results were in line with the findings of the study conducted on mustard inhibitors [24]. *Msti* not only affected the growth of aphids, but they also had a significant role in controlling the spotted alfalfa aphid reproduction. The mechanism of interaction between the trypsin inhibitors and insects includes a positive regulation where trypsin inhibitors can irreversibly react with the trypsin that is secreted by the insect’s gut to form a complex of inactivity and irreversibility, which inhibits the absorption of nutrients by insects and passes on the signals to insects for feed inhibition [42]. This dual-effect by the inhibitors minimizes the utilization of plant protein by insects, which subsequently reduces the nutritional supply or the loss of digestive function, and then inhibits the growth and development of insects, eventually causing their death. Feeding cabbage moth larva with SKTI reduced their body mass by 25%, whilst inhibiting food digestion and nutrients absorption. However, the effect on the mortality of larva was non-significant [41]. The activity of trypsin in the midgut of *Heliothis zea* (*Helicoverpa armigera*) and beet armyworm (*Spodoptera exigua*) was significantly increased by the intake of trypsin inhibitors [43]. Another approach is negative regulation, similar to that of mammals, in which pancreatic enzyme secretion increases when the pancreas gets larger, and a disproportionate intake of trypsin inhibitors inhibits the mammalian digestion and absorption by the regulation of the hormone cholecystokinin. An analysis revealed that the intestinal trypsin function might have inhibited protein supply after the ingestion of *Msti*. However, the specific mechanism still has to be elucidated, especially the activity of aminopeptidase, which seems to be affected by the inhibition of trypsin and trypsin-like enzymes. Both *Msti* recombinant purified proteins at a concentration of 800 μg/mL inhibited the activity of four proteases in aphids, and ultimately interfered with the normal protein hydrolysis and metabolism in aphids. This led to retarded growth, development, and reproduction, which finally caused death of the aphids.

Combating insect pests is a common issue for plant geneticists, breeders, and entomologists. Multiple insect-resistant genes have been successfully applied to develop transgenic insect-resistant varieties. However, the role of exogenous genes still has uncertain effects on the plant itself [44,45], although they have insect resistance. Hence, an acceptable remedy would be to focus on the development of new types of bio-pesticides with the potential to control pests using genes conferring resistance in the form of proteins.

## 5. Conclusions

We note that two of the Kunitz trypsin inhibitor genes (*Msti-94* and *Msti-16*) affected the growth, development, and reproduction of spotted alfalfa aphids by reducing the cellular enzyme activity. Both the *Msti* genes likely affected the metabolic pathways by altering enzymatic activity after 72 h of oral exposure (feeding). *Mtsi* genes show promise as a route for developing bio-pesticides against the spotted alfalfa aphid, wherein its integration into IPM programs will require additional research into their specific roles in reducing aphid survival and reproduction.

## Figures and Tables

**Table 1:** Formula of the artificial diet for the spotted alfalfa aphids.

Reagent Name	Dosage (or Concentration)
CuCl_2_·2H_2_O	0.02 g
FeCl_3_·6H_2_O	1.1 g
MnCl_2_·4H_2_O	0.04 g
ZnSO_4_·H_2_O	0.085 g
MgSO_4_·7H_2_O	0.123 g
K_2_HPO_4_·3H_2_O	1.5 g
Biotin	0.01 g
Folic acid	0.05 g
Lactochrome	0.025 g
Calcium pantothenate	0.05 g
Ammonium sulfate (NH_4_)_2_ SO_4_	0.025 g
Pyridoxine hydrochloride	0.025 g
Casein hydrolysate	2 g
Yeast powder	2 g
Honey	10 g
Saccharose	30 g
Choline chloride	0.05 g
Inose	0.05 g
Niacin	0.01 g
Ascorbic acid	0.1 g
Para-aminobenzoic	0.01 g
ddH_2_O	500 mL
KOH/NaOH (0.5 M)	0.5 mol/L

**Table 2:** List of primers used for the RT-qPCR.

Genes	Primers (5′-3′)
*Msti-64*	CTTCCTTTCTCTCTTAGCGTTG; GCATCAGGGTTACGGATTAC
*Msti-86*	CCTTCTGGGCTTCACTTCA; TACAACGACACTGAGGAGGG
*Msti-94*	CACCATACCAGGAATAAGTCC; GACCACCAATACCAACACAAG
*Msti-95*	GGTGTAGAAATAACTGGTGGCA; GTCCAACATAAGCACAATCTCC
*Msti-14*	ACTCCTATGGCAGCAGAAGA; CACTCCTATTGTCCCTATCACC
*Msti-16*	GGGTGTAGAAGGTAATCCAAGT; CAGGACAGAAAGAAAGCACA

**Table 3:** Selected *Msti-16* and *Msti-94* genes for their gene sequences.

Genes	Gene Sequences
*Msti-16*	**GGATCC**ATGAAAACCTCACTCTTAGCATTTTCCACCATCTTTTTAGCCTT CATTTGCAAAACTATTGCAGCACCTGAACCAGTTCTTGACATTTCAGGC AAACAAGTGACAACTGGTGTAAAATACTATATTTTACCAGTCATAAGA GGTAAAGGTGGTGGTTTAACAGTTGCAAACCATGGTGAAAACAACCAA ACATGTCCCCTTTATGTTGTTCAAGAGAAGCTTGAAGTAAAGAATGGTG AAGCAGTTACTTTCACACCTTATAATGCTAAACAAGGTGTGATTCTAAC TTCTACTGATCTCAATATTAAGTCCTTTGTAACAAAAACTAAATGTCCTC AAACACAAGTTTGGAAGCTTCTTAAAGAGTTGACAGGGGTGTGGTTTTT AGCTACAGGGGGTGTAGAAGGTAATCCAAGTATGGCAACTGTTGGTAA TTGGTTTAAGATTGAGAAAGCTGATAAAGATTATGTGCTTTCTTTCTGTC CTGCTGAAGCTTGCAAATGTCAAACTTTGTGTAGGGAACTAGGGTTGTT TGTTGATGATAAGGGAAATAAGCACTTAGCTCTTAGTGATCAAATTCCA TCATTTAGGGTTGTGTTTAAAAGGGCTTAA**CTCGAG**
*Msti-94*	**GGATCC**ATGAAGCATCTTTTATCACTAACCCTTTCCTTCTTCATCTTTGT TTTCATCACCAATCTTTCACTAGCTACTTCAAATGATGTTGAGCAAGTAT TGGACATAAATGGTAACCCCATTTTCCCAGGTGGTCAATACTACATTTT GCCAGCACTTCGTGGCCCCGGAGGAGGAGGAGTAAGATTAGGAAGAA CCGGTGATTTAAAGTGTCCAGTTACCGTCTTACAAGATCGTAGAGAAG TCAAGAATGGTCTACCAGTGAAATTCACCATACCAGGAATAAGTCCTG GTATAATTTTCACTGGTACACCACTTGAGATCGAGTACACGAAGAAAC CTAGTTGCGCTGAATCAACAAAATGGTTAATATTTGTTGATAATGTTATT GGAAAAGCTTGTGTTGGTATTGGTGGTCCTGAAAATTACCCTGGTGTGC AAACATTGAGTGGCAAATTTAATATTCAGAAACATGCATCTGGATTTG GTTATCAGCTAGGGTTTTGTGTTACGGGGTCTCCTACTTGTTTGGATATT GGAAGGTTTGATAATGATGAAGCTGGAAGACGTTTGAATTTGACTGAA CATGAGGTTTATCATGTTGTGTTTGTTGATGCAGCTACTTATGAAGCTGA GTATATTAAGTCTGTTGTTTAA**CTCGAG**

The underlined bases in the table above represent the restriction sites for BamH I (GGATCC) and Xhol I (CTCGAG).

**Table 4:** Amino acid sequences of the selected *Msti-16* and *Msti-94* genes.

Genes	Amino Acid Sequences
*Msti-16*	MKTSLLAFSTIFLAFICKTIAAPEPVLDISGKQVTTGVKYYILPVIRGKGGGLTV ANHGENNQTCPLYVVQEKLEVKNGEAVTFTPYNAKQGVILTSTDLNIKSFV TKTKCPQTQVWKLLKELTGVWFLATGGVEGNPSMATVGNWFKIEKADKD YVLSFCPAEACKCQTLCRELGLFVDDKGNKHLALSDQIPSFRVVFKRA*
*Msti-94*	MKHLLSLTLSFFIFVFITNLSLATSNDVEQVLDINGNPIFPGGQYYILPALRGP GGGGVRLGRTGDLKCPVTVLQDRREVKNGLPVKFTIPGISPGIIFTGTPLEIEY TKKPSCAESTKWLIFVDNVIGKACVGIGGPENYPGVQTLSGKFNIQKHASGF GYQLGFCVTGSPTCLDIGRFDNDEAGRRLNLTEHEVYHVVFVDAATYEAEYIKSVI*

* Represents sequence translation termination site.

**Table 5:** SDS-PAGE protein electrophoresis gel formula.

Reagent	10% Separation Gel
ddH_2_O	1.4 mL
Tris Buffer (pH 8.8)	1.3 mL
30% Acrylamide	1.7 mL
10% SDS (Sodium dodecyl sulfate)	0.05 mL
10% APS (10% Ammonium persulfate solution)	0.05 mL
TEMED (Tetramethylethylenediamine)	0.003 mL
Propanetriol	0.5 mL

**Table 6:** Sequence homology of the selected candidate genes (*Msti-16* and *Msti-94*) obtained from the National Center for Biotechnology Information (NCBI).

Candidate Genes	Identity	Number (NR)	Description
*Msti-16*	95%	XM_003620121.2:49-660	Kunitz trypsin inhibitor
*Msti-94*	95%	AF526372.1:1773-2423	Kunitz trypsin inhibitor

NR (Gene database in NCBI).

## Supplementary Materials

The following are available online at https://www.mdpi.com/2075-4450/10/6/154/s1, Figure S1: Step-wise process of spotted alfalfa aphids reared with an artificial diet. (A) Healthy active spotted alfalfa aphid; (B) Aphid’s artificial diet; (C) feeder with aphids sealed from the bottom with a sponge; (D) artificial diet poured on the first layer of Parafilm M^®^; (E) sealed with another layer of Parafilm M^®^ from the top; (F) aerial view of aphids feeding. Table S1: Equation of different enzyme curve along with regression value; Table S2: Enzyme activities assays of aphids on different concentration of feed with *Msti-94* recombinant protein; Table S3: Enzyme activities assays of aphids on different concentration of feed with *Msti-16* recombinant protein; Table S4: Enzyme activities assays of ahpids on different feed with recombinant purified protein.

## Abbreviations

Msti	*Medicago sativa* trypsin inhibitors
EI	Enzyme-Inhibitor; PI: Protease inhibitors
CpTi	Cowpea trypsin inhibitors
TI	Trypsin inhibitors
CTP	Chymotrypsin inhibitors
CPI	Cysteine proteinase inhibitor
STI	Soybean trypsin inhibitors
MTI	Mustard trypsin inhibitors
ELISA	Enzyme-linked immunosorbent assay
RNA	Ribonucleic acid
DNA	Deoxyribonucleic acid
cDNA	Complementary DNA
mRNA	Messenger RNA
SDS	Sodium dodecyl sulfate
SDS-PAGE	Sodium dodecyl sulfate-Polyacrylamide gel electrophoresis
PBS	Phosphate-buffered saline
HRP	Horseradish peroxidase
DEGs	Differentially expressed genes
IPTG	Isopropyl β-D-1-thiogalactopyranoside
KTI	Kunitz type trypsin inhibitors
kDa	Kilodalton
RT-qPCR	Quantitative real time polymerase chain reaction
OD	Optical density
MBP	Maltose Binding Protein
GST	Glutathione *S*-transferase
His	Histidine
BApNA	*N*-α-benzoyl-L-arginine ρ-nitroanilide
L-Leu-pNA	L-Leucine-*p*-nitroanilide
