# Supplementary material for: Inhibitory Effects of Plant Trypsin Inhibitors Msti-94 and Msti-16 on Therioaphis trifolii (Monell) (Homoptera: Aphididae) in Alfalfa
Source: Insects. 2019 May 30;10(6):154. doi: 10.3390/insects10060154 (PMC6627745; doi:10.3390/insects10060154)
Supplement: Supplementary file 1 [file insects-10-00154-s001.pdf]

insects

MDPI

Article

Inhibitory Effects of Plant Trypsin Inhibitors *Msti-94* and *Msti-16* on *Therioaphis trifolii* (Monell) (Homoptera: Aphididae) in Alfalfa

Hailong Zhao [1,2,†], Hidayat Ullah [1,3,†], Mark Richard McNeill [4], Guilin Du [5], Kun Hao [1], Xiongbing Tu [1,*] and Zehua Zhang [1,*]

1 State Key Laboratory for Biology of Plant Diseases and Insect Pests, Institute of Plant Protection, Chinese Academy of Agricultural Sciences, Beijing 100193, China; zhao.hailong@live.com (H.Z.); shabkadar@yahoo.com (H.U.); haokun8611@foxmail.com (K.H.)
2 College of Plant Protection, Shenyang Agricultural University, Shenyang 110161, China
3 Department of Agriculture, The University of Swabi, Anbar-23561, Swabi, Khyber Pakhtunkhwa, Pakistan
4 AgResearch, Lincoln Research Centre, Christchurch 8140, New Zealand; mark.mcneill@agresearch.co.nz
5 National Animal Husbandry Service, Beijing 100125, China; caasdgl@163.com
* Correspondence: xbtu@ippcaas.cn (X.T.); zhangzehua@caas.cn (Z.Z.)
† These authors contributed equally to this study.

Received: 6 March 2019; Accepted: 24 May 2019; Published: date

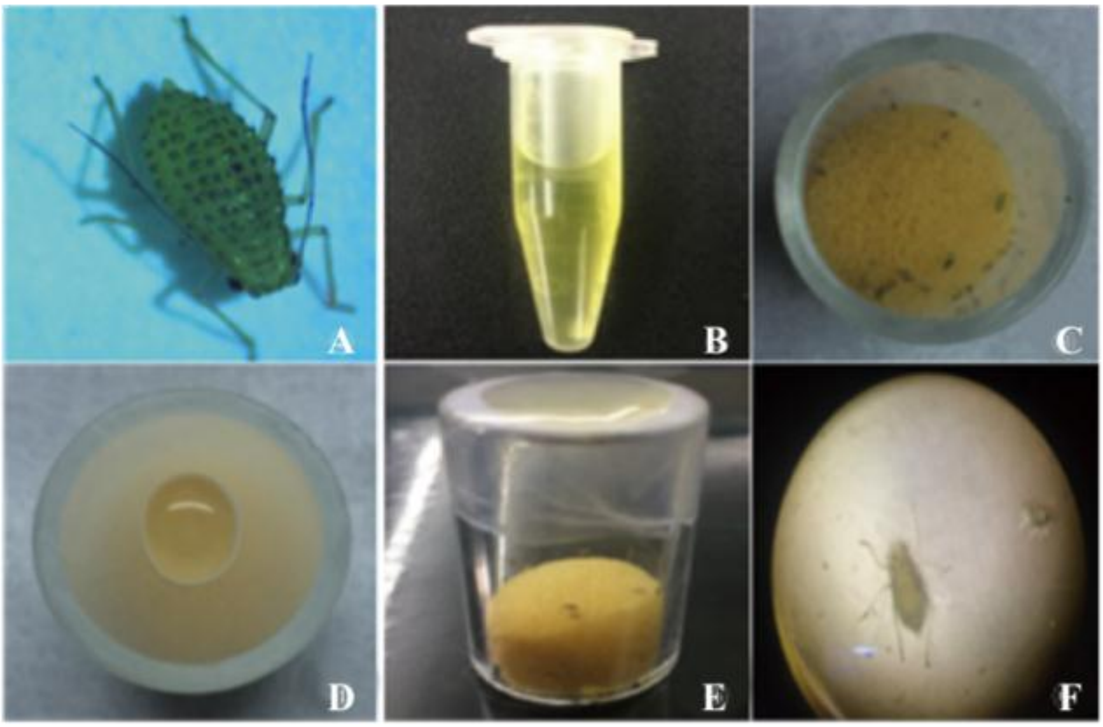

Figure S1. Step-wise process of spotted alfalfa aphids reared with an artificial diet. (A) Healthy active spotted alfalfa aphid; (B) Aphid's artificial diet; (C) feeder with aphids sealed from the bottom with a sponge; (D) artificial diet poured on the first layer of Parafilm M; (E) sealed with another layer of Parafilm M from the top; (F) aerial view of aphids feeding.

Table S1. Equation of different enzyme curve along with regression value.

Enzyme	Equation	R^2
Total proteinase	y=0.0025x+0.0822	0.9932
Trypsin	y=0.0002x+0.0642	0.9951
Chymotrypsin	y=0.0397x+0.0934	0.9910
Aminopeptidase	y=0.0239x+0.0964	0.9914

Table S2. Enzyme activities assays of aphids on different concentration of feed with *Msti-94* recombinant protein.

Concentration (μg/mL)		Enzyme (U/mg)			
		Total Proteinase	Trypsin	Chymotrypsin	Aminopeptidase
**2000**	Control	164.56±8.80a	2265.33±40.21a	11.58±0.39a	19.23±0.93a
	Msti-94	159.81±22.84a	2136.01±141.26a	9.78±0.77b	17.77±1.26a
3000	Control	169.59±10.90a	2252.93±62.07a	11.55±0.69a	19.33±0.98a
	Msti-94	156.52±14.45a	2096.15±177.30b	8.30±0.55c	17.87±0.50a
4000	Control	170.84±14.57a	2376.21±306.04a	11.07±0.54a	18.03±1.16a
	Msti-94	148.53±24.97b	1775.56±58.95c	8.37±0.59c	16.04±0.97b
5000	Control	173.19±7.49a	2247.07±148.25a	10.57±0.76a	18.32±0.20a
	Msti-94	114.00±5.71c	1561.47±109.28d	6.29±0.32d	11.74±1.19c
6000	Control	172.84±2.21a	2229.82±163.16a	10.81±0.75a	18.30±1.72a
	Msti-94	67.42±13.96d	981.57±99.38e	4.14±0.74e	8.67±1.22d

Data described in terms of "mean value ± standard error" at 0.05 level of significant ranged by Duncan analysis with SPSS 20.0.

Table S3. Enzyme activities assays of aphids on different concentration of feed with *Msti-16* recombinant protein.

Concentration (μg/mL)		Enzyme (U/mg)			
		Total Proteinase	Trypsin	Chymotrypsin	Aminopeptidase
**2000**	Control	174.66±14.33a	2314.24±107.64a	11.28±0.71a	7.39±1.15e
	Msti-16	151.58±17.38b	2169.88±302.27b	10.85±0.85a	17.58±0.90b
3000	Control	184.12±6.61a	2197.65±73.79b	10.88±0.50a	22.04±0.40a
	Msti-16	145.72±14.54b	2181.57±214.15b	9.63±0.42b	16.18±0.53bc
4000	Control	181.33±5.05a	2312.06±95.73a	10.77±0.75a	17.83±0.18b
	Msti-16	137.38±5.70c	1614.91±932.63c	8.37±0.59c	15.18±0.78c
5000	Control	171.99±22.47a	2251.09±291.00a	10.49±0.84a	18.26±1.60b
	Msti-16	115.00±7.77d	1260.64±304.80d	6.41±0.76d	11.91±1.01d
6000	Control	170.10±6.90a	2187.60±95.50a	10.90±1.04a	17.46±1.80b
	Msti-16	72.63±20.83e	913.07±176.25e	5.04±0.51e	11.26±1.15d

Data described in terms of "mean value ± standard error" at 0.05 level of significant ranged by Duncan analysis with SPSS 20.0.

Table S4. Enzyme activities assays of ahpids on different feed with recombinant purified protein.

Treatments (800 μg/mL)	Enzyme			
	**Total Proteinase (U/mg)**	**Trypsin (U/mg)**	**Chymotrypsin (U/mg)**	**Aminopeptidase (U/mg)**
Control	235.64±9.04a	2903.18±168.20a	13.42±0.67a	22.06±0.51a
Msti-94	139.06±19.25b	1761.64±150.45b	9.13±1.14b	15.86±0.84b
Msti-16	137.18±16.80b	1687.94±104.17b	8.81±0.94b	15.11±0.83b
